# Parental characteristic patterns associated with maintaining healthy physical activity behavior during childhood and adolescence

**DOI:** 10.1186/s12966-016-0383-9

**Published:** 2016-05-06

**Authors:** Soyang Kwon, Kathleen F. Janz, Elena M. Letuchy, Trudy L. Burns, Steven M. Levy

**Affiliations:** Ann & Robert H. Lurie Children’s Hospital of Chicago Stanley Manne Children’s Research Institute, 225 E Chicago Ave. Box 157, Chicago, IL 60611 USA; University of Iowa Department of Health and Human Physiology, 130 Field House, Iowa City, IA 52242 USA; University of Iowa Department of Epidemiology, 145 N. Riverside Drive, S418 CPHB, Iowa City, IA 52242 USA; University of Iowa Department of Preventive and Community Dentistry, 801 Newton Road, Dental Science Building, Iowa City, IA 52242 USA

**Keywords:** Latent class, Father’s role in child physical activity, Sports participation, Moderate- to vigorous-intensity physical activity

## Abstract

**Background:**

Parental characteristics that influence child physical activity (PA) behavior often co-occur. An analytic approach that considers these co-occurring patterns can help researchers better understand the overall context of parental influence. The study aims were to: (1) identify diverse patterns of the relationships among parental characteristics, (2) examine the influence of these parental patterns on child sport participation and moderate-to vigorous-intensity PA (MVPA) trajectories during childhood and adolescence, and (3) examine whether family support mediates the influence of the parental patterns on child sport participation and MVPA trajectories.

**Methods:**

We used data from 408 Iowa Bone Development Study cohort families (97 % Caucasians; 65 % mothers with a 4-year college degree). From ages 5 to 19 years, the cohort participated in seven accelerometry assessments, reported sports participation every 6 months, and reported perceived family support for PA at age 15. Parents reported family income, education level, and regular PA participation in high school and adulthood. Structural equation modeling was conducted to identify the latent classes represented among these parental characteristics. Sex-adjusted multivariable logistic regression analyses were conducted to predict sports participation trajectories and MVPA trajectories by latent class and family support.

**Results:**

Three parent latent classes were identified: higher family socioeconomic status (SES) and regular PA in both high school and adulthood by both the father and mother (Group 1); lower family SES and regular PA in high school by the father (Group 2); and lower family SES and no regular PA in high school by the father (Group 3). Sex-adjusted ORs of the “drop-out from sports participation” pattern for the children in Groups 1 and 2, compared to Group 3, were 0.38 (95 % CI = 0.20, 0.72) and 0.51 (95 % CI = 0.26, 1.00), respectively. Sex-adjusted ORs of the “decreasing from moderate MVPA” pattern for the children in Groups 1 and 2, compared to Group 3, were 0.29 (95 % CI = 0.11, 0.75) and 1.16 (95 % CI = 0.40, 3.37), respectively. Adding family support to the logistic regression model only slightly changed the ORs.

**Conclusions:**

The findings from this study suggest that among lower SES families, the father’s role may be important to promote youth to sustain sports participation.

## Background

Parents can influence their children’s physical activity (PA) behavior by controlling the structure of their children’s time and resources, such as through buying sports equipment or transporting a child to sport lessons and activities [1]. However, the recently released Midcourse Report of the U.S. Physical Activity Guidelines [1] notes that there is little research to clearly substantiate how and how much parents influence child PA. The report prioritizes the need for more research on this issue [1]. A model of parental influence on child PA [2] has been suggested to illustrate that father’s and mother’s PA modeling may influence child PA directly, or that this association may be mediated by family support. Many studies have attempted to identify individual parental measures associated with child PA, such as family socioeconomic status, parent PA behavior, and family support for child PA [3, 4]. However, these studies have often used an analytic approach that does not consider the potential co-occurring patterns of the measured parental characteristics, although those parental characteristics have been shown to be related to each other [5, 6]. Furthermore, that relationship could be heterogeneous within a study population. More advanced analytic approaches, such as latent class analysis, can provide a data reduction process to detect the presence of meaningful unmeasured constructs (latent classes) that can illustrate the different patterns of the relationships among measured parental variables. Identifying these distinct patterns can provide a better understanding of the context of the parental influence on child PA behavior. The current study was conducted based on findings from our two previous analyses of Iowa Bone Development Study (IBDS) data: one analysis [7] found that, although the majority of youth (67.0 % of the cohort) decreased moderate- to vigorous-intensity PA (MVPA) during childhood and adolescence, a subsample of youth (18.1 % of the cohort) was consistently active throughout childhood and adolescence (14.9 % were consistently inactive). The same analysis [7] found that about four in five children joined organized sports and that about half of them dropped out over time, while the other half maintained their participation. Our other analysis [8] reported that youth who reduce their moderate- to vigorous-intensity PA (MVPA) levels are more likely to become obese in young adulthood, as compared to youth who are consistently active throughout childhood and adolescence. With this health consequence in mind, herein we explored whether heterogeneous parental characteristic constructs could explain why some children maintain healthy PA behavior and others do not. This study had three aims: to identify diverse patterns of relationships among parental factors using latent class analysis, to examine the influence of the parental patterns on child sport participation (maintenance vs. drop-out) and MVPA (maintenance vs. decrease) trajectories during childhood and adolescence, and to examine whether family support mediates the influence of parental patterns on child sport participation and MVPA trajectories.

## Methods

### Participants

Secondary analysis was conducted using the IBDS datasets. The IBDS is an ongoing longitudinal study of bone health during childhood, adolescence, and young adulthood. Participants are a subset of the Iowa Fluoride Study birth cohort that included 1882 families recruited from eight Iowa hospital postpartum wards between 1992 and 1995 [9]. The IBDS used rolling admission to allow Iowa Fluoride Study members to participate in any follow-up examinations. Child cohort members participated in baseline accelerometer assessments between 1998 and 2002 at age 5 years, as well as six follow-up assessments at approximate ages of 8, 11, 13, 15, 17, and 19 years. In our previous MVPA trajectory analysis, [7] we included 537 participants who completed at least three PA assessments over time. For the current analysis, we excluded participants whose fathers did not report PA in high school and adulthood (*n* = 129; 24.0 %), because, unlike the PA data for mothers (missing *n* = 18; 3.4 %), the proportion of missing PA data for fathers was too large to include in the latent class analysis with an assumption of missing at random. Family income distribution was similar in families with and without PA data for fathers (chi-square *p* = 0.56). Of the 408 families who were included in the analysis, 96.8 % were Caucasian and 48 % of the participating children were female. Informed parental consent and child assent were obtained from all participants. The University of Iowa Institutional Review Board (IRB) approved the IBDS.

### Parental characteristics assessment

During study visits at child age 11 or 13 years, fathers and mothers reported their weekly frequencies of leisure-time PA in the previous year as well as during their high school years: “How often did you play sports or exercise during the past year [in your high school years]? Never, once a month, 2 to 3 times a month, once a week, more than once a week.” Engaging in PA more than once a week was considered to be regular PA. Parents also reported family income and mother’s education level in a mailed-in demographic survey in 2007 (around child age of 13 years). Family income and mother’s education level were dichotomized based on their median value.

### Perceived family support assessment

A subsample of child participants completed the Choices Questionnaire at ages 13 (*n* = 281) and 15 years (*n* = 314). The questionnaire contained five individual family support items: “In the last 7 days, how often has a member of your household (1) encouraged you to do PA or play sport, (2) done PA or played sports with you, (3) provided transportation to a place where you can do PA or play sports, (4) watched you participate in PA or sport, and (5) told you that you are doing well in PA or sport?” Five response options of never, once, sometimes, almost every day, and every day, were given and scored from 1 to 5, respectively. The family support scale was developed and validated for the Amherst Study [10]. In our pilot reliability test among 52 IBDS participants, the internal consistency for the five family support items was 0.85 [11]. The mean family support score at each of the two assessments was dichotomized based on the median for the sample. Because a larger sample completed the family support assessment at age 15 compared to at age 13, age 15 data were selected as a family support indicator for analysis. For those without age 15 family support data, age 13 family support data were used. An IBDS report [11] showed that the family support score remained moderately stable over time. As a previous meta-analysis [4] reported variation in the effect size for each of the five individual family support behaviors, each of the five individual family support item scores was dichotomized based on the median for the sample to explore the effect of each of the five individual support behaviors.

### Child sports participation and physical activity assessment

Detailed information on the assessment and definition of organized sports participation is described in our previous publication [7] Briefly, to assess organized sports participation, two versions of a physical activity questionnaire (PAQ) were used. The first version (proxy-PAQ), which was designed for the parent to report his or her child’s activities, was used for children younger than age 11 years. The proxy-PAQ asked about sports participation: “Did your child participate in any of the following organized sports during the past 6 months? Baseball, basketball, soccer, gymnastics, dance, and swimming.” Respondents were also allowed to report other organized sports that were not listed. The second version (self-PAQ) was adopted from the PAQ for older children (PAQ-C) [12] and PAQ for adolescents (PAQ-A) [13] and used for children at age 11 years or older. The self-PAQ contained a list of 20+ sports and inquired about the frequency of sports participation in the past 7 days and whether or not the sports activity was organized (defined as “a coached sport or lessons” in the PAQ). If a respondent reported participating in at least one sport and marked it as “organized”, the respondent was categorized as “participating in organized sport.” Using longitudinal sports participation data, our previous work [7] identified three heterogeneous trajectory patterns of sports participation over childhood and adolescence: no sports participation, drop-out from sports participation, and consistent sports participation.

Child PA was assessed using ActiGraph accelerometers at approximately 5, 8, 11, 13, 15, 17, and 19 years of age (accelerometer model 7164 at ages 5 to 13, GT1M at age 15, and GT3X+ at ages 17 and 19; Pensacola, FL). The detailed procedures for accelerometer data collection are described in our previous publications [14, 15]. Briefly, child participants received an accelerometer via mail during the autumn season (September to November) and were asked to wear the accelerometer during waking hours for four consecutive days (including one weekend day) at ages 5 and 8, and for five consecutive days (including both weekend days) at ages 11 to 19. For the accelerometry data reduction process, accelerometers were considered as having not been worn if a period of 60 consecutive minutes of zero accelerometry counts (with an allowance for two non-zero interruptions) was encountered in the accelerometry data array. Only the accelerometry data from participants who wore an accelerometer for at least 8 h per day and for at least 3 days at each assessment were used [16] MVPA (minutes/day) was daily accumulated number of minutes with 2296 or greater accelerometry counts per minute [17, 18]. Using the longitudinal MVPA data, our previous work [7] identified four heterogeneous trajectory patterns of MVPA over childhood and adolescence: consistently inactive, consistently active, decreasing from moderate MVPA, and substantially decreasing from high MVPA.

### Statistical analysis

Frequency analyses were conducted for the dichotomized family support variable and the six dichotomized parental characteristic variables, including family income, mother’s education, father’s regular PA in high school, father’s regular PA in adulthood, mother’s regular PA in high school, and mother’s regular PA in adulthood. To achieve the first aim, latent class analysis was conducted to detect the presence of latent classes that described the patterns of the six parental characteristic variables. The initial latent class analysis assumed five latent classes; analysis was repeated while decreasing the number of latent classes. Model fit was evaluated using the Bayesian Information Criterion (BIA). Based on the posterior maximum likelihood, each participant was assigned to one of the latent classes.

To achieve the second aim, cohort members who followed the “no sport participation” pattern (*n* = 48) and the “consistently inactive” pattern (*n* = 43) were excluded, resulting in 360 participants for the sport participation analysis and 365 participants for the MVPA analysis. Chi-square tests were conducted to test the associations of the latent classes with family support (higher vs. lower), sports participation trajectories, and MVPA trajectories. Multivariable logistic regression analysis was conducted to estimate the probability of the “drop-out from sports participation” pattern as compared to the “consistent sports participation” pattern, including predictors, sex and the latent class. MVPA multinomial logistic regression analysis was conducted to predict the probability of “decreasing from moderate MVPA” and “substantially decreasing from high MVPA”, compared to “consistently active”, including predictors, sex and the latent class. For the third aim, the analyses were repeated with the addition of the dichotomized family support variable as a predictor. The effect of each of the five family support items was also tested. Additional multivariable logistic regression analysis was conducted with the family support variable assessed at age 13 years to examine whether use of family support data assessed at age 13 versus at age 15 produced different results and found no significant difference. The interaction effects of sex × latent class, latent class × family support, and sex × family support were tested and found no significant interaction. A significance level was set at 0.05.

## Results

**Fig. 1:**
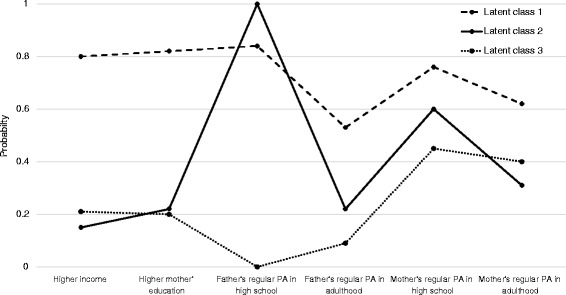
Probability of six parental characteristic variables according to latent class. PA, physical activity

Table 1 shows the distributions of the main exposure variables and a latent class variable representing the combination of family SES and parental PA behavior. Three latent classes were identified: higher family socioeconomic status (SES) and regular PA engagement in high school and adulthood by both mother and father (“high SES”; 47.9 %); lower family SES and regular PA in high school by the father (“father’s high school PA”; 37.6 %); and lower family SES and no regular PA in high school by the father (“father’s no high school PA”; 14.5 %). The likelihood of regular PA in adulthood by the mother tended to be higher in the “high SES” class than in the other two classes. The mean posterior probability was 87 % for the “high SES” class, 87 % for the “father’s high school PA” class, and 87 % for the “father’s no high school PA” class. The probabilities of each of the six parental characteristics for each of the latent classes are illustrated in Fig. 1. As shown in Table 2, while family support levels were similar between the “high SES” group and the “father’s high school PA” group, the “father's high school PA” group tended to have lower family support (*p* = 0.08). Chi-square analyses showed that the three latent classes were significantly associated with two sport participation trajectory patterns and three MVPA trajectory patterns.

**Table 1:** Frequency distribution of parental characteristics, perceived family support, and the parental characteristic latent classes

	*n* (%)
Total number of families	408 (100)
Annual family income in 2007	
< $80,000	196 (49.4)
≥ $80,000	184 (47.9)
Mother’s education level	
<4-year college degree	261 (65.1)
≥4-year college degree	140 (34.9)
Father’s regular PA in high school	
No	90 (22.2)
Yes	315 (77.8)
Father’s regular PA in adulthood	
No	261 (65.1)
Yes	140 (34.9)
Mother’s regular PA in high school	
No	140 (34.8)
Yes	262 (65.2)
Mother’s regular PA in adulthood	
No	211 (53.0)
Yes	187 (47.0)
Family support for child PA	
Lower	208 (55.8)
Higher	165 (44.2)
Parental characteristic latent classes	
Higher family SES and regular PA engagement in high school and adulthood by both mother and father	192 (47.1)
Lower family SES and regular PA in high school by the father	152 (37.3)
Lower family SES and no regular PA in high school by the father	64 (15.7)

*PA* physical activity, *SES* socioeconomic status

**Table 2:** Distributions of the parental characteristic latent classes according to family support, child sports participation trajectories, and child moderate- to vigorous-intensity physical activity (MVPA) trajectories

	Higher family SES and regular PA engagement in high school and adulthood by both father and mother	Lower family SES and regular PA in high school by the father	Lower family SES and no regular PA in high school by the father	*P*-value
		*n* (%)		
Total	192 (47.1)	152 (37.3)	64 (15.7)	
Family support (*n* = 408)				0.08*
Lower (*n* = 208)	90 (43.3)	79 (38.0)	99 (18.7)	
Higher (*n* = 165)	85 (51.5)	62 (37.6)	18 (10.9)	
Missing (*n* = 35)	17 (48.6)	11 (31.4)	7 (20.2)	
Sports participation trajectory (*n* = 360)^a^				0.01
Consistent sports participation (*n* = 194)	111 (57.2)	65 (33.5)	18 (9.3)	
Drop-out from sports participation (*n* = 166)	75 (45.2)	59 (35.5)	32 (19.3)	
MVPA trajectory^a^ (*n* = 365)				<0.01
Consistently active (*n* = 63)	45 (71.4)	12 (19.1)	6 (9.5)	
Decreasing from moderate MVPA (*n* = 243)	104 (42.8)	99 (40.7)	40 (16.5)	
Substantially decreasing from high MVPA (*n* = 59)	30 (50.8)	23 (39.0)	6 (10.2)	

*MVPA* moderate- to vigorous-intensity physical activity, *SES* socioeconomic status

*Chi-square *p*-value was calculated excluding the missing data

^a^Sport participation and MVPA trajectories were reported in Kwon et al. [7]

A sex-adjusted logistic regression model showed that youth with “higher SES” were less likely than youth with “father’s no high school PA” to be in the “drop-out from sport participation” pattern, as opposed to the “consistent sports participation” pattern (odds ratio [OR] = 0.38; 95 % confidence interval [CI] = 0.20, 0.72; Table 3), and to be in the “decreasing from moderate MVPA” pattern, as opposed to the “consistently active” pattern (OR = 0.29; 95 % CI = 0.11, 0.75; Table 4). Youth with “father’s high school PA” were less likely than youth with “father’s no high school PA” to be in the “drop-out from sports participation” pattern (OR = 0.51; 95 % CI = 0.26, 1.00). When family support was added to the model, the strength of the associations of “higher SES” with the “drop out from sports participation” trajectory (OR = 0.38; 95 % CI = 0.18, 0.79) and with the “decreasing from moderate MVPA” trajectory (OR = 0.30; 95 % CI = 0.11, 0.87) remained at a similar level. Lower family support was associated with the “drop-out from sports participation” pattern (OR = 0.34; 95 % CI = 0.22, 0.54) and the “decreasing from moderate MVPA” pattern (OR = 0.51; 95 % CI = 0.27, 0.95). The “substantially decreasing from high MVPA” pattern was not significantly associated with the parental characteristic latent classes or family support. The slightly different results for “decreasing from moderate MVPA” and “substantially decreasing from high MVPA” as shown in Table 4 could be because the “substantially decreasing from high MVPA” group had a smaller sample size, and/or because, given that 88 % of participants in the “substantially decreasing from high MVPA group were boys, the strong sex effect might have weakened the estimated association between “substantially decreasing from high MVPA” and the latent class variable.

**Table 3:** Odds ratios (ORs) and 95 % confidence intervals (CIs) of “drop-out from sports participation” vs. “consistent sports participation”^a^ from multivariable logistic regression models of sports participation trajectories

	Logistic model without a family support variable	Logistic model with a family support variable
Predictor	OR (95 % CI)	OR (95 % CI)
Sex: males vs. females	0.79 (0.52, 1.20)	0.76 (0.48, 1.20)
Latent class: higher family SES and regular PA engagement in high school and adulthood by both father and mother vs. lower family SES and no regular PA in high school by the father	0.38 (0.20, 0.72)	0.38 (0.18, 0.79)
Latent class: lower family SES and regular PA in high school by the father vs. lower family SES and no regular PA in high school by the father	0.51 (0.26, 1.00)	0.51 (0.24, 1.09)
Family support: higher vs. lower	--	0.34 (0.22, 0.54)

*PA* physical activity, *SES* socioeconomic status

^a^Sport participation trajectories were reported in Kwon et al. [7]

**Table 4:** Odds ratios (ORs) and 95 % confidence intervals (CIs) from multinomial logistic regression models of moderate- to vigorous-intensity physical activity (MVPA) trajectories

	Logistic model without a family support variable		Logistic model with a family support variable	
Predictor	OR (95 % CI) of “decreasing from moderate MVPA” vs. “consistently active”^a^	OR (95 % CI) of “substantially decreasing from high MVPA” vs. “consistently active”^a^	OR (95 % CI) of “decreasing from moderate MVPA” vs. “consistently active”^a^	OR (95 % CI) of “substantially decreasing from high MVPA” vs. “consistently active”^a^
Sex: male vs. female	0.23 (0.12, 0.43)	2.81 (1.01, 7.82)	0.19 (0.09, 0.37)	1.94 (0.67, 5.59)
Latent class: higher family SES and regular PA engagement in high school and adulthood by both father and mother vs. lower family SES and no regular PA in high school by the father	0.29 (0.11, 0.75)	0.72 (0.21, 2.45)	0.30 (0.11, 0.87)	0.49 (0.14, 1.81)
Latent class: lower family SES and regular PA in high school by the father vs. lower family SES and no regular PA in high school by the father	1.16 (0.40, 3.37)	1.96 (0.52, 7.44)	1.15 (0.36, 3.65)	1.52 (0.38, 6.07)
Family support: higher vs. lower	--	--	0.51 (0.27, 0.95)	1.07 (0.50, 2.31)

*MVPA* moderate- to vigorous-intensity physical activity, *PA* physical activity, *SES* socioeconomic status

^a^MVPA trajectories were reported in Kwon et al. [7]

In the analysis of individual family support items, the ORs of being in the “drop-out from sports participation” trajectory for each of the five family support items ranged from 0.32 to 0.56. Watching a child play PA or sports (OR = 0.32) and telling a child that he or she is doing well in PA or sports (OR = 0.33) were more strongly associated with the “consistent sport participation” pattern, as compared to encouraging a child to participate in PA or sports (OR = 0.56). The ORs of being in the “decreasing from moderate MVPA” trajectory for each of the five family support items ranged from 0.52 to 0.93.

Sex-specific multivariable logistic regression analysis revealed that the positive associations of “father’s high school PA” and high family support of “consistent sports participation” were similar among males and among females. The association between lower family support and the “decreasing from moderate MVPA” pattern was stronger among males (OR = 0.38; 95 % CI = 0.18, 0.83) than among females (OR = 0.87; 0.27, 2.85).

## Discussion

This study identified heterogeneous relationships among six of the parental characteristics under investigation. As expected, one identified relationship pattern showed higher family SES clustering with regular PA in high school as well as in adulthood for both parents, while another pattern showed lower family SES clustering with no regular PA of fathers in high school or in adulthood. One distinct pattern that represented 37.6 % of the study population was higher engagement in regular PA of fathers during high school among lower SES families. As expected, youth in the “higher SES” latent class were more likely than youth in the “father’s no high school PA” latent class to follow consistent sports participation and consistently active trajectory patterns as opposed to following decreasing patterns. Interestingly, youth in the “father’s high school PA” latent class tended to follow a consistent sports participation pattern, suggesting the importance of the father’s role in promoting the child’s consistent participation in sports throughout childhood and adolescence among lower SES families. Higher family support was associated with consistent sports participation and consistently active patterns without weakening the association between the latent class variable and the sports participation and MVPA trajectories, which suggests that family support could be an independent determinant rather than a mediator.

Our finding of healthier PA patterns among youth in higher SES families clustering with higher PA engagement of parents is consistent with previous studies [3, 4] and was not a surprise. However, to the best of our knowledge, no studies have reported that there are distinct patterns of PA engagement history during high school among fathers within lower SES families, or that these distinct patterns are associated with their children’s continuous participation in organized sports. The mechanism underlying how father’s PA history affects the child’s maintenance of participation in organized sports is largely unknown. The findings from this study indicate that this association cannot be explained by father’s PA modeling or family support. A qualitative study [19] reported that one way in which parents facilitate PA in their child is by prioritizing PA. Of the few qualitative studies on the father’s role in promoting child PA, a study by Zahra et al. [20] reported that, in terms of priority, the mother seemed more involved in the academic development of the child, whereas the father was more interested in child PA, and suggested that the father plays a key role in prioritizing and promoting child PA. The current decreasing trend in the number of U.S. youth who participate in organized sports [21] suggest that it is important for future studies to examine how father’s regular PA history influences consistent participation in organized sports among youth within lower SES families, who we know are at a higher risk for unhealthy PA behaviors and various physical inactivity-related health outcomes. Overall, few research studies have included fathers to examine the father’s impact on their child’s PA behavior, [20] partly because fathers tend to be more difficult to recruit into research studies [22–24]. The Australian Healthy Dads Healthy Kids study, one of the few randomized controlled trials that targeted fathers and children, demonstrated the importance of the father’s role in the PA behaviors of their children [25]. More research to understand the father’s role in children’s PA behavior is warranted.

Based on our findings, it appears that family support is independently associated with child’s healthy PA behavior, rather than mediating the influence of parental characteristics on child PA. Our finding of the positive association between family support and consistent PA participation is consistent with a recent meta-analysis, [4] and confirms that family support for child PA is an important strategy to sustain healthy PA behavior. In terms of a specific family support behavior that is effective, the meta-analysis [4] suggests that encouragement is the strongest predictor of child’s healthier PA behavior. However, our study found that rather than having a family member encourage a child to participate in PA or sports, having a family member watch a child exercise and/or tell a child that he or she is doing well in PA or sports were more strongly associated with maintenance of sport participation and MVPA. Watching and noting success indicate that the PA or sport participation is already occurring. Therefore, these activities could be capturing a positive family culture of PA or sport participation and would be most valuable for maintaining current PA or sport participation as compared to beginning new PA.

This study is unique in that it applied an innovative analytic approach to a relatively large, well-described cohort to better understand the context of parental influence on child PA behavior development. This study is also unique in its use of objectively-measured PA from childhood to young adulthood. However, several limitations should be acknowledged. First, excluding those youth participants without father’s PA data might have introduced selection bias. Although we did not collect data on the residential status of both parents, it is likely that the youth who were included in the analysis lived with both parents, which limits the generalizability of the study results to youth who do not live with both parents. Second, the study population included mostly higher SES rural Caucasians. Thus, the results may not be generalizable to other populations. Third, because only a subsample completed the family support assessment at age 15, we used the data collected at age 13 for those without family support data at age 15, which could have caused some misclassification. However, in the sensitivity analysis for those with only a family support assessment at age 13, we found similar results for the association between family support and the outcomes. Fourth, this secondary data analysis did not allow us to separately examine the effects of parents’ participation in high school sports vs. community sports. Lastly, the multivariable logistic regression models did not take into account various unmeasured PA determinants, such as school PA opportunities and built environmental factors. Therefore, the observed association could have been biased by unmeasured confounding.

## Conclusions

Applying a latent class analytic approach, this study demonstrated heterogeneous relationships among parental characteristic variables that are known correlates of child PA behavior. The findings from this study suggest that among lower SES families, the father’s role may be important in promoting youth to sustain organized sports participation.

## Ethics, consent and permissions

Informed consent and assent were obtained from all participants.

## Consent to publish

I have obtained consent to publish from the participants (or legal parents or guardians for children) to report individual patient data.

## Abbreviations

IBDS: Iowa Bone Development Study
MVPA: moderate- to vigorous-intensity physical activity
PA: physical activity
SES: socioeconomic status
